# 199. Prospective Observational Cohort Of Transition to Oral Antibiotics in Persons Who Inject Drugs with Invasive Infections

**DOI:** 10.1093/ofid/ofab466.401

**Published:** 2021-12-04

**Authors:** Sophia Lewis, Laura Marks, Liang Stephen, Nathan Nolan, Michael Durkin

**Affiliations:** 1 Barnes Jewish Hospital, St. Louis, Missouri; 2 Washington University in St. Louis, St. Louis, MO; 3 Washington U Sch of Med, St. Louis, Missouri; 4 Washington University, St. Louis, Missouri

## Abstract

**Background:**

Persons who inject drugs (PWID) are at increased risk of invasive bacterial infections. Increasing data supports the efficacy of transition to oral antibiotic therapy to complete treatment of invasive bacterial infections including osteomyelitis and endocarditis. The aim of this study is to evaluate the impact of transition to oral antibiotics on a prospective observational cohort of PWID.

**Methods:**

We prospectively analyzed PWID admitted 2/2020 - 2/2021 at Barnes-Jewish Hospital in St. Louis with osteomyelitis, endocarditis, epidural abscesses or septic arthritis. All patients were offered multidisciplinary support during their inpatient hospitalization including addiction medicine consultation and medications for opioid use disorder, if appropriate. Health coaches and case managers met with patients during their hospitalization and followed patients for up to 90 days after discharge. Patients were offered the option of transition to oral antibiotics if they were not able to complete recommended IV antibiotics. Patients discharged on oral antibiotics were offered post-discharge infectious diseases follow-up. Antibiotic adherence was documented by health coaches through phone out-reach. We collected data on demographics, comorbidities, microbiologic data, antibiotic selection, mortality and readmission rates. We compared 90-day readmission rates between PWID who completed IV antibiotics inpatient and those who discharged early with oral antibiotics.

**Results:**

Of 166 PWID, 61 completed IV antibiotics inpatient (37%) while 105 were discharged with oral antibiotics (63%). Causative pathogens were not significantly different between inpatient IV vs oral antibiotics; MSSA (34.4% vs 35.2%, p= 0.92), MRSA (34.4% vs. 28.6%, p=0.43), or streptococcal species (26.6% vs. 24.8%, p=0.85). Of patients discharged on oral antibiotics 7.6% had documented non-adherence to therapy, 23% had unknown adherence and 67% had documented adherence. There was no significant difference in all-cause 90-day readmission rates (p=0.819) (Figure 1).

All-cause readmissions by antibiotic strategy

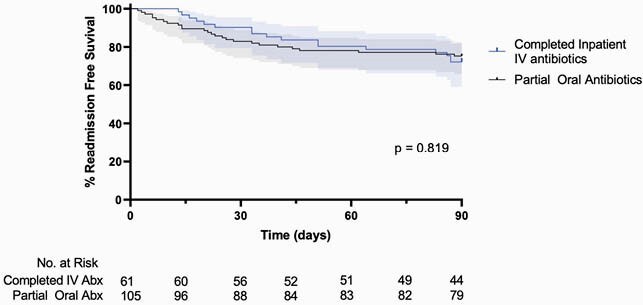

**Conclusion:**

Oral antibiotic regimens provided similar efficacy to IV antibiotics in our prospective cohort analysis of PWID.

**Disclosures:**

**All Authors**: No reported disclosures

